# Families With Violence Exposure and the Intergenerational Transmission of Somatization

**DOI:** 10.3389/fpsyt.2022.820652

**Published:** 2022-02-23

**Authors:** Jennifer Glaus, Dominik A. Moser, Sandra Rusconi Serpa, Sondes Jouabli, Fiorella Turri, Kerstin J. Plessen, Daniel S. Schechter

**Affiliations:** ^1^Division of Child and Adolescent Psychiatry, Department of Psychiatry, Lausanne University Hospital (CHUV) and University of Lausanne, Lausanne, Switzerland; ^2^Faculty of Psychology, University of Geneva, Geneva, Switzerland; ^3^Department of Psychiatry, Faculty of Medicine, University of Geneva, Geneva, Switzerland; ^4^Department of Child and Adolescent Psychiatry, New York University Grossman School of Medicine, New York, NY, United States

**Keywords:** intergenerational transmission, somatization, violence exposure, post-traumatic stress disorder, child psychopathology, children, mothers, traumatic events

## Abstract

**Introduction:**

Adults who have histories of childhood trauma have been noted to display greater somatization, dissociative symptoms and affect dysregulation. What happens in the parent-child relationship when those traumatized children become parents? A potential link to somatization in the child has been suggested by several prior studies. Children who have early attachment disturbances had more physical complaints if their mothers displayed less maternal sensitivity during observed parent-child interactions. Yet, the intergenerational link between maternal and child somatization has not been sufficiently explored in a longitudinal study in order to understand the potential impact of maternal trauma history and related psychopathology on subsequent child somatization and psychopathology.

**Methods:**

This paper examined prospective, longitudinal data of 64 mother-toddler dyads (mean age = 2.4 years, SD = 0.7) who were later studied when children had a mean age of 7 years. Mothers with and without histories of interpersonal violence (IPV; physical/sexual abuse and/or family violence exposure) were included. Mothers with IPV histories were oversampled. Linear and Poisson regression models were used to test the associations between maternal IPV-related post-traumatic stress disorder (PTSD) with maternal somatization severity when children were toddlers, and between maternal somatization and maternal interactive behaviors with child somatization by maternal report and clinician-rated assessment at school-age.

**Results:**

Maternal PTSD severity was significantly associated with increased maternal somatization severity (*p* = 0.031). Maternal somatization severity during the child's early childhood predicted both maternal report of child somatization (*p* = 0.011) as well as child thought problems (*p* = 0.007) when children were school-aged. No association was found between maternal somatization and child-reported psychopathology. The study did not find that maternal alexithymia, caregiving behaviors or child exposure to violence contributed significantly to the model examining the association between maternal and child somatization.

**Conclusion:**

The results are in line with the hypothesis of intergenerational transmission of somatization in the context of IPV and related maternal PTSD during formative early development. We interpret this as an expression of psychological distress from mother to child, as maternal trauma and pathology affect the caregiving environment and, thus, the parent–child relationship. The authors conclude with a discussion of implications for parent–infant and early childhood intervention.

## Introduction

Somatization refers to the expression of physical symptoms resulting from psychological stress and as such is frequently associated with child trauma, such as community and domestic violence exposure and child maltreatment (1–6). When prolonged and warranting of professional attention, somatization in this context may lead to unnecessary medical visits, evaluative procedures and treatments that increase iatrogenic risk (1). Significant somatization as is found in relation to child trauma often also contributes to decreased school attendance and academic performance, peer-related social functioning, eating, sleeping, emotion regulation, and the ability to experience pleasure. Somatization in the absence of any plausible biomedical explanation for the physical symptoms experienced, becomes a form of psychopathology that can additionally exacerbate and be exacerbated by mood and anxiety disorders (7–9). And prominent somatoform symptoms during childhood have been associated with greater psychiatric comorbidity, such as depression and anxiety disorders, by early adulthood (10–12). As a category of psychopathology, somatization or “somatoform” disorders are not only impairing to the individual child or adolescent as described above, but they also have an impact on the healthcare system. The numerous in-patient stays and out-patients visits in order to rule-out somatic illness and establish a diagnosis that explains the patient's somatic distress are associated with enormous costs (13). Somatoform disorders have a prevalence rate in youth and adults estimated to vary between 10 and 25% both in primary care (14) and in the general population (15).

In a meta-analysis of 34 studies of functional psychiatric illness (i.e., somatoform disorders), two factors that emerged across the majority of studies were the illness following an external trigger or “suggestion” and abnormal body awareness (16).

More recently, the notion of somatization as a bodily memory and/or representation of what is unmentalized—in other words, without apparent awareness of mental states of self and other, has been associated with trauma as a form of “embodiment” of traumatic memory and related emotional reactions (17, 18). This notion is particularly salient if the trauma occurred early in life, such as during the first 4–5 years of life, before the development of social cognition, self-regulation and adequate language skills to communicate one's experience and suffering (19, 20). The term “Developmental Trauma Disorder” used by van der Kolk et al. (21) encompasses this among other phenomena in such individuals with early-onset, chronic traumatization.

Over the past decade research on complex post-traumatic stress disorder (cPTSD) as a consequence of an exposure to early-onset trauma has been conducted. cPTSD is defined as a group of symptoms similar to an highlighted PTSD with additional clusters of symptoms, including emotional dysregulation, negative self-cognitions, and interpersonal hardship (22). Neuroimaging research has demonstrated that among individuals suffering from early-onset trauma with chronic cPTSD, many if not most will experience dissociation and somatization involving dampening of neural circuits that are responsible for the integration of cognitive and affective aspects of memory traces (23, 24). This observation has led to the development of a dissociative subtype of PTSD in the DSM-5, which has been demonstrated to be highly associated with somatization (25). The late 19th, early 20th century French psychologist who developed the concept of psychological dissociation Pierre Janet posited that because of the threat to psychic integrity, somatic symptoms such as pain, numbness, dizziness, fainting, and nausea would develop (26). Neuroimaging research has attempted to understand these phenomena with several models under consideration, all involving the anterior insula, dorsal-lateral prefrontal cortex, subgenual anterior cingulate, amygdala and hippocampus (26–28).

Somatoform disorders have been associated with low educational levels, cultural idioms of distress, and with socio-economic disadvantage, in which individuals—such as newly immigrants who might have experienced multiple traumatic events before and during migration, may be more likely to express somatic symptoms (29–31). This may well be because these individuals at higher risk for somatization are less likely to have acquired metacognitive skills (32–34). Such individuals may come from a culture in which somatization is a frequent expression of psychic distress (32, 33, 35, 36).

Insecure attachment has been associated with related concepts, including alexithymia (inability to identify and verbalize emotions)—which marks impairment in mentalization, among patients with severe somatoform disorders (37). Moreover, the association between alexithymia and somatization is well-established (35, 36).

When children who have experienced developmental trauma (i.e., early life stress) in an impoverished caregiving environment become parents, they may contribute to a perpetuation of trauma exposure to their children, either directly because of emotional dysregulation and poor impulse control, and/or indirectly due to blind-spots for such risk factors with respect to their romantic partners. Thus, their offspring may show higher levels of somatization than offspring of caregivers who are non-traumatized and who do not generally have the same frequency of attachment disturbances (38, 39). Pathological somatization in the wake of early life stress is particularly important to address due to the fact that the expression of developmental traumatic stress through bodily symptoms that are split off from psychological awareness of associated mental states (emotions, thoughts, fantasies, fears, and desires), does not permit the individual suffering to integrate traumatic memory traces into a coherent narrative such that somatic symptoms become more automatic and repetitive. And thus somatization often does not permit the individual to reconsolidate those memory traces underlying these symptoms so as to render those memory traces less distressing (40). Similarly for this reason, a parent who remains psychologically blind to the traumatic origins of their suffering and who undergoes unnecessary medical testing, procedures and other interventions that can secondarily traumatize the individual, will not be able adequately to help their children with an awareness of the link between their mental states and physical symptoms, leading to intergenerational transmission, and potentially to a vicious cycle of retraumatization (40, 41). Children who have disturbances of early attachment, indeed show elevated somatization particularly in the context of low maternal sensitivity (38). The literature also reflects the possibility that individuals with early developmental trauma may process sensory input including psychological stress and pain, differently than others, due to a combination of factors that include sensory overload and somatic memory traces of abuse (42). An absence of feedback in the form of sensitive responsiveness and help with emotional regulation may similarly play an important role in validating painful emotional experiences within the attachment relationship (43–45). In one review of the literature, several familial risk factors for the development of somatoform disorders in youth were identified, including: somatization of parents, psychopathology of close family members, dysfunctional family climate, and traumatic experiences in childhood (46). Somatoform disorders are also associated with other psychiatric disorders in children, most commonly those of anxiety and depressive disorders (46).

Yet the intergenerational link has not been sufficiently and directly enough explored in a longitudinal study to date to understand the role of maternal trauma history and related psychopathology that might affect the mother-child relationship during formative development of emotion identification and regulation during the first years of life. In turn, the present study is novel in that it examines how the impact on the early mother-child relationship may impact subsequent child somatization (1, 46, 47) and other child psychopathology at school-age—this, while also considering maternal alexithymia, maternal sensitive caregiving, and child exposure to violence. The aims of this study were therefore to:

(1) Examine the associations between maternal history of interpersonal violence related PTSD (IPV-PTSD) and maternal somatization during Phase 1 (1–3.5 years after birth of the child);
(2) Investigate longitudinally the effect of maternal somatization when children were ages 1–3.5 years old on child somatization and child psychopathology when children were 5–9 years old (both maternal and child-reported);
(3) Assess the role of maternal caregiving behaviors, alexithymia and child exposure to family violence and/or treatment on the association between maternal and child somatization and psychopathology.

## Materials and Methods

### Participants and Procedure

The data of the present paper stem from the Geneva Early Childhood Stress Project (GECS-Pro), a longitudinal study that included two phases and investigates the role of maternal IPV-PTSD on their children. The study was conducted between 2010 and 2018. Flyers were posted at different locations, including the Geneva University Hospital, domestic violence agencies, community centers and shelters, the latter to oversample mothers exposed to domestic violence. The protocol was then briefly explained to interested mothers. Upon participation and when thought necessary by the clinician and the supervisor of the study, feedback was offered to participants.

Inclusion criteria were as follows: biological mothers who were willing to consent to their child's participation in the study and who were able to speak French; and their children aged between 12 and 42 months. We excluded mothers with active substance abuse or psychotic disorder, and fathers were not invited to participate in the study because some mothers were living under order of protection or otherwise in anonymous shelters for battered women.

The procedure has been described in detail (48). Briefly, we conducted Phase 1 when children were 12–42 months-old, which is a formative period for the development of emotion regulation in the context of the parent-child relationship. Phase 2 was conducted when children were school-aged (5–9 years old), as children in this age-range are expected to have sufficient self-regulation of emotion in order to be able to learn and socialize.

A total of 84 mother-child dyads participated in Phase 1. Eighty percent also participated in Phase 2 (*n* = 67 dyads). Dropouts from Phase 1–2 did not significantly differ from mothers and children who did participate in Phase 2 in terms of age, gender, SES, or number of maternal traumatic life-events. Three mother-child dyads were further excluded due to incomplete data on child psychopathology. The final sample used to examine the intergenerational transmission of somatization between mothers and their children consisted of 64 children (mean age = 2.4 [1.0–3.7] years) of mother with or without a diagnosis of IPV-PTSD.

The study was approved by the Institutional Ethics' Committee of the Geneva University Hospitals and is in accordance with the Helsinki Declaration (49). All participants were informed about the study and gave written consent.

### Measures

#### Maternal Variables

We collected information on maternal age and socio-economic status (SES) during Phase 1, using the Geneva Socio-Demographic Questionnaire (50), which included the Largo index (51) to measure socio-economic status.

We assessed mothers for maternal IPV-PTSD using the structured interview the Clinical Administered PTSD Scale (CAPS) (52) in Phase 1. The version of the CAPS that was used included 30 items corresponding to the DSM-IV diagnosis for PTSD, and yielded a total symptom severity score. It is the gold standard in PTSD assessment, with high sensitivity (90%) and specificity (95%), as well as a Cronbach's alpha coefficient of 0.97 (53). Mothers with a score higher than 40 and/or who fulfilled DSM-IV criteria for lifetime PTSD were categorized as having a diagnosis of lifetime PTSD.

We measured maternal somatization using the somatization subscale of the widely-used Symptom Checklist-90-Revised inventory (SCL-90-R) (54, 55) during Phase 1. This instrument assesses a wide variety of psychopathological symptoms, including somatization among nine other categories of psychopathology. Each item is rated on a 5-point scale of distress (0 = never, to 4 = extremely). The total score for the somatization subscale was used for the present study, as a continuous variable. Good reliability and validity have been demonstrated for the SCL-90-R (56).

Maternal intrusive/controlling behavior was assessed in Phase 1 *via* the Care-Index (57–59). Coded observations were videotaped during a 5-min bout of mother-child play taken from a 25-min interaction protocol (60), with individual subscales concerning the mother and concerning the child, respectively. For the present analysis, we used data representing coded observations of maternal behavior. Specific aspects of interactions, including the mother's ability to comfort the child, the receptivity to mother-child turn-taking, shared pleasure, joint attention, non-verbal and verbal negotiation, and reciprocal communication, were rated by a coder. Seven dimensions of interactive behavior were taken into account: facial and vocal expression, position and body contact, expression of affection, turn-taking, control, and choice of activity. Three types of caregiving behaviors are assessed along continuous scales: sensitive, controlling and unresponsive caregiving behavior. The three maternal behavior scales range from 0 to 14 (for example for controlling behavior: from 0 = not being at all controlling, to 14 = being extremely controlling). Two experienced clinical psychologists independently rated the videos. Inter-rater reliability of the Care-index was excellent (ICC = 0.92), as well as of the three maternal behavior scales (ICC sensitive = 0.88; ICC controlling = 0.86; ICC unresponsive = 0.85) (61).

We additionally evaluated maternal alexithymia in Phase 1 using the French version of the Toronto Alexithymia Scale (TAS-20) (62). This is a well-validated instrument composed by 20 items resulting in three subscales, including ability to identify feelings, ability to describe feelings, and externally oriented thinking. For the present analysis, we only used the “ability to identify” scale, resulting in a continuous variable. Overall internal consistency of the TAS-20 French version was acceptable (Cronbach's alpha = 0.74) (61).

#### Children's Variables

Children's age was, as for mothers, assessed using the Geneva Socio-Demographic Questionnaire (50) in Phases 1 and 2.

Concerning child somatization in Phase 2, we used the maternal reported subscale score of the well-validated Child Behavior Checklist (CBCL) (63). This instrument evaluates psychopathology in children during the past 6 months. It is composed by 113 items and is scored on a three-point Likert scale (0 = absent, 1 = occurs sometimes, and 2 = occurs often). We categorized the items into nine scales, including anxious/depressed, somatic complaints, withdrawn, social problems, thought problems (e.g., obsessive thoughts, self-harm, hallucinations, ruminations, strange behavior, and nervous twitching), attention problems, obliviousness to rules and to authority, and other problems (e.g., overeating, sleeping more, thumb suck, enuresis). For child somatization, we considered the somatic complaints scale, which includes the following symptoms as defined by the DSM-IV: aches, headaches, nausea, eye problems, skin problems, stomachaches, vomiting. The total CBCL, as well as the somatic complaints subscale showed a good internal consistency (α = 0.90; α = 0.62, respectively).

We also assessed child-reported mental disorder symptoms in children in Phase 2, using the French version of the semi-structured interview Schedule for Affective Disorders and Schizophrenia for School-aged Children—Epidemiologic version (K-SADS-E) (64). The reliability of this instrument has been tested previously, showing high kappa coefficients ranging from 0.84 for major depressive disorder (MDD) to 0.86 for separation anxiety disorder (SAD) (65). Because only certain modules of the interview were used, we took into account the number of symptoms of the following mental disorders: SAD, general anxiety disorder (GAD), MDD, attention deficit/hyperactivity disorder (ADHD), PTSD, conduct disorder (CD), and oppositional defiant disorder (ODD).

We further assessed child exposure to family violence and/or maltreatment, using the Geneva Child Exposure to Violence Questionnaire [CETV; (48)], adapted from the violence subscale of the Child Exposure to Domestic Violence Scale (CEDV; α = 0.78) (66). This instrument is composed of 10 items and evaluates the frequency a child witnessed the violence on a 4-points-scale (from 0 = “never” to 3 = “almost always”), as well as who was the main actor (e.g., father of the child, family member). The CETV showed a good internal consistency (α = 0.75). For the present study, we created a dichotomized variable (i.e., 0 = “non-exposed”; 1 = “exposed”).

### Statistical Analysis

We standardized continuous independent variables and created descriptive data for demographic characteristics, maternal variables (i.e., total symptom severity score for PTSD, history of traumatic events, somatization, caregiving behaviors, and alexithymia), as well as for child somatization, psychopathology, and exposure to family violence. Then, *t*-tests were used to compare the differences between mother with and without IPV-PTSD for somatic complaints (both maternal and child somatization). To evaluate the association between IPV-PTSD and somatization in mothers in Phase 1 (aim 1) we used multiple linear regression models adjusted for age and SES in mothers. We then performed Spearman correlations for the relations between maternal somatization in Phase 1 and child psychopathology in Phase 2 (aim 2), since the number of symptoms in children was not following a linear distribution. For statistically significant correlations, we tested the associations using Poisson regression models, since the number of symptoms in children were following a Poisson distribution. We used multiple linear regression models to test the associations between maternal somatization in Phase 1 and development of maternal reported child somatization in Phase 2. For both types of associations, we computed six models of increasing complexity: Model 1 was unadjusted; Model 2 was adjusted for SES and child age and sex in Phase 2; Model 3 was further adjusted for maternal caregiving behaviors; Model 4 was further adjusted for child exposure to family violence and/or maltreatment; Model 5 was further adjusted for maternal alexithymia; and finally Model 6 was further adjusted for maternal IPV-PTSD. Furthermore, in order to understand better the mechanism of the association between maternal somatization and child somatization (aim 3), we performed additional linear regression models for the link between maternal somatization and caregiving behaviors, alexithymia and child exposure, as well as for the associations between maternal controlling behavior, alexithymia and child exposure with thought problems in children. Additionally, due to the finding of a link between maternal somatization and alexithymia, two complementary *post-hoc* regression models with three predictors (maternal somatization and alexithymia, and the interaction of the two) were performed. The first model aimed to test the interaction between maternal somatization and alexithymia upon child somatization, and the second one upon thought problems in children. Using G^*^Power 3.1 (67) we tested *post-hoc* statistical power for such analyses for our study and found that given a moderate effect size of f2 = 0.15 such an interaction would be significant in 84% of the cases.

We conducted the statistical analyses using the Statistical Analysis System (SAS Institute Inc., Cary, NC, USA) version 9.4 for Windows.

## Results

### Sample Characteristics

Results of the sample characteristics are presented in Table 1. Mothers were 35 years old (SD = 6.02) on average, and at the assessment in Phase 1, 62.5% of them experienced IPV-PTSD in their lifetime. Among those, 69% of mothers with PTSD were physically abused as children, 29% sexually abused and nearly 64% exposed to domestic violence as children (60). The mean score for maternal somatization was 6 (SD = 4.37). Mothers with IPV-PTSD reported a higher mean score for somatization compared to mothers without IPV-PTSD (mean = 6.9, SD = 4.91; mean = 4.4, SD = 2.75, respectively; *p* = *0.011*). A total of 28 out of 64 (44%) children in our Phase 2 sample were girls, with a mean age of 7 years old (SD = 1.13). Children had a mean score of 2 (SD = 2.01) for maternal-reported somatic complaints. With respect to maternal somatization, children of mothers with IPV-PTSD reported higher somatic complaints among their children, compared to non-PTSD mothers (mean = 2.4, SD = 2.32; mean = 1.3, SD = 1.33, respectively; *p* = *0.014*). Concerning child psychopathology, higher scores were found for the maternal-reported scales of aggressive behavior, “other problems” and anxious/depressed symptoms. We found that children reported on average 1.4 symptoms per psychiatric disorder (i.e., the total mean of the number of symptoms children reported on the K-SADS for each disorder), with SAD, MDD, and PTSD being the most frequent, and the behavioral disorders, namely CD and ODD being the least frequent. Moreover, 12.5% of children had already been exposed to family violence and/or maltreatment in their life.

**Table 1:** Sample characteristics of children and mothers (*n* = 64 dyads).

	**Mean (SD)**	**Minimum–maximum**
**Maternal variables in Phase 1**		
Age, years	34.59 (6.02)	22–47
SES	5.07 (1.91)	2–9
CAPS total symptom severity score	59.42 (35.34)	16–129
Somatization	5.95 (4.37)	0–17
Caregiving behaviors^*^		
Sensitive behavior	5.16 (1.36)	2–7
Controlling behavior	3.09 (1.68)	0–7
Unresponsive behavior	2.64 (1.67)	0–6
Alexithymia	14.68 (5.32)	7–27
**Child variables in Phase 2**		
**Sex, % (** ***n*** **)**		
Girls	43.75 (28)	–
Boys	56.25 (36)	–
Age, years (Phase 1)	2.40 (0.70)	1.00–3.67
Age, years (Phase 2)	7.02 (1.13)	4.67–10.00
**Somatization (maternal-reported)**		
Somatic complaints	1.97 (2.01)	0–10
**Child psychopathology (maternal-reported)**		
Anxious/depressed	5.11 (4.78)	0–18
Social problems	2.31 (2.17)	0–12
Aggressive behavior	9.02 (5.19)	0–21
Thought problems	0.86 (1.21)	0–5
Withdrawn	2.95 (2.39)	0–10
Attention problems	4.30 (3.47)	0–14
Rule-breaking behavior	1.72 (1.33)	0–6
Other problems	6.97 (4.31)	0–20
**Number of symptoms (child-reported)**		
Separation anxiety disorder	1.95 (1.61)	0–5
General anxiety disorder	1.16 (1.20)	0–4
Major depressive disorder	1.52 (1.51)	0–6
Attention Deficit/Hyperactivity Disorder	1.13 (1.37)	0–4
Post-traumatic Stress Disorder	2.35 (3.31)	0–13
Conduct disorder	0.61 (1.05)	0–4
Oppositional defiant disorder	0.78 (1.0)	0–3
**Exposure to family violence and/or maltreatment**		
Yes, % (*n*)	12.50 (8)	–
No, % (*n*)	73.44 (47)	–

*SES, Socio-Economic Status; CAPS, Clinical Administered PTSD Scale*.

* *19 missing for caregiving behavior variables*.

### IPV-PTSD and Somatization in Mothers (Aim 1)

After adjustment for maternal age and SES, mothers with IPV-PTSD were significantly more likely to report greater maternal somatization in Phase 1 compared to non-PTSD mothers (β = 1.31, 95 confidence interval [CI: 0.12–2.50], *p* = *0.031*).

### Associations Between Maternal Somatization With Child Somatic Complaints and Other Psychopathology (Aim 2)

**Figure 1:**
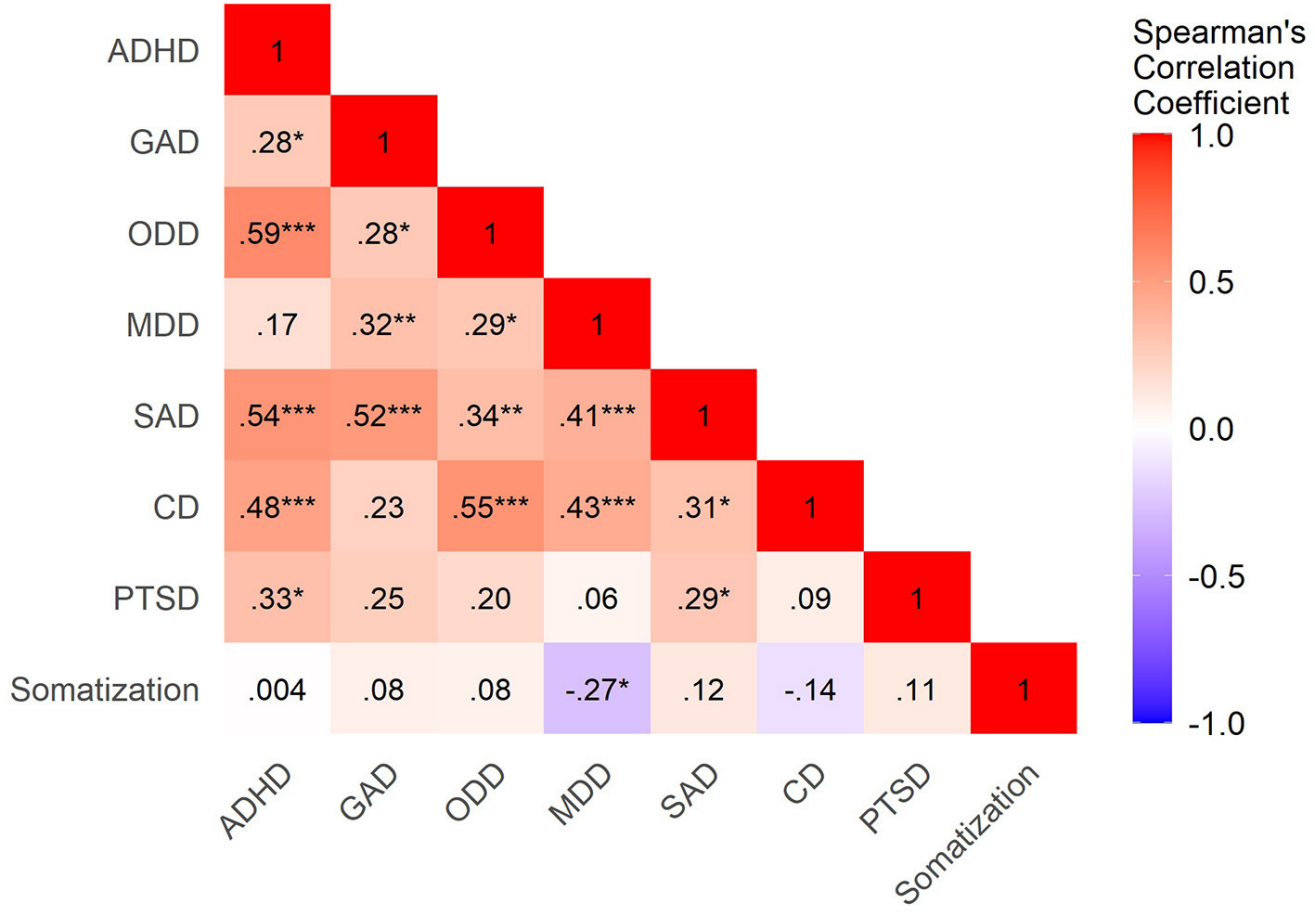
Heatmap of correlations between maternal somatization in Phase 1 and child psychopathology in Phase 2. ADHD, Attentional Deficit/Hyperactivity Disorder; GAD, Generalized Anxiety Disorder; ODD, Oppositional Defiant Disorder; MDD, Major Depressive Disorder; SAD, Separation Anxiety Disorder; CD, Conduct disorder; PTSD, Post-Traumatic Stress Disorder. Correlation coefficients are indicated in the squares. Blue means negative correlations, whereas red are positive correlations. ***p < 0.001; **p < 0.01; *p < 0.05.

**Figure 2:**
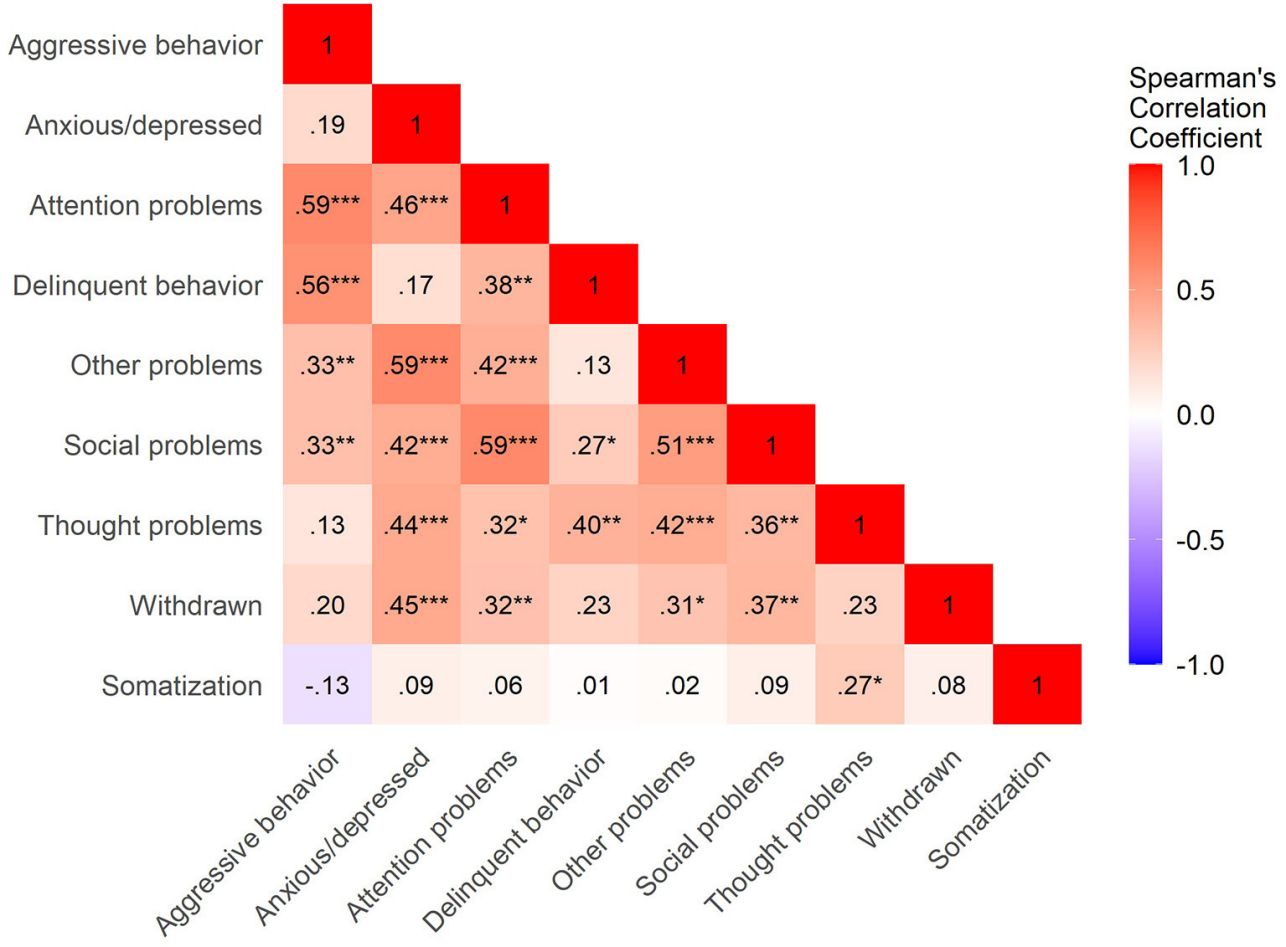
Heatmap of correlations between maternal somatization in Phase 1 and child psychopathology (maternal-report) in Phase 2. Correlation coefficients are indicated in the squares. Blue means negative correlations, whereas red are positive correlations. ***p < 0.001; **p < 0.01; *p < 0.05.

Concerning the correlations between maternal somatization in Phase 1 and child psychopathology in Phase 2, we found that the number of MDD symptoms in the child at school-age was, in the end, the only form of measured psychopathology correlated to maternal somatization (c.f., Figure 1). Specifically, more severe maternal somatization was, surprisingly, negatively correlated to the number of child MDD symptoms on the K-SADS; whereas, the correlations between maternal somatization and child symptoms of other forms of clinician-rated child psychopathology were not statistically significant. Additionally, we found a correlation between maternal somatization in Phase 1 and maternal-reported child thought problems on the CBCL in Phase 2 (c.f., Figure 2). Therefore, we further tested only the associations between maternal somatization and the following measures: child somatization, the number of MDD symptoms and thought problems in the children (c.f., Table 2).

**Table 2:** Associations between maternal somatization in Phase 1 with child somatic complaints and child psychopathology in Phase 2 (*n* = 64).

	**Child somatization (maternal report)**		**MDD (child self-report)**		**Thought problems (maternal report)**	
**Maternal variables**	**β**	***p***	**β**	***p***	**β**	***p***
**Model 1**						
Somatization	**0.74**	***0.047***	**−0.32**	***0.047***	**0.37**	***0.040***
**Model 2**						
Somatization	**0.78**	***0.041***	**−0.33**	***0.046***	0.23	*0.217*
**Model 3**						
Somatization	**1.26**	***0.015***	−0.41	*0.075*	0.31	*0.227*
Controlling behavior	0.90	*0.089*	−0.14	*0.472*	0.23	*0.395*
Sensitive behavior	0.93	*0.118*	−0.20	*0.411*	0.17	*0.577*
Unresponsive behavior	0.60	*0.223*	−0.27	*0.171*	−0.10	*0.683*
**Model 4**						
Somatization	**1.90**	***0.003***	−0.25	*0.296*	**0.92**	***0.007***
Controlling behavior	0.57	*0.339*	−0.22	*0.313*	0.08	*0.788*
Sensitive behavior	0.42	*0.529*	−0.29	*0.264*	0.24	*0.481*
Unresponsive behavior	0.31	*0.577*	−0.28	*0.169*	−0.17	*0.491*
Child exposure to violence	1.00	*0.375*	0.32	*0.440*	0.93	*0.067*
**Model 5**						
Somatization	**1.85**	***0.008***	−0.19	*0.465*	0.70	*0.068*
Controlling behavior	0.47	*0.461*	−0.23	*0.336*	−0.67	*0.113*
Sensitive behavior	0.34	*0.630*	−0.32	*0.226*	0.20	*0.583*
Unresponsive behavior	0.16	*0.791*	−0.30	*0.170*	**−0.77**	***0.027***
Child exposure to violence	1.18	*0.315*	0.45	*0.282*	**1.72**	***0.017***
Alexithymia	0.20	*0.626*	−0.02	*0.886*	**0.87**	***0.007***
**Model 6**						
Somatization	**1.79**	***0.011***	−0.25	*0.341*	0.50	*0.168*
Controlling behavior	0.49	*0.446*	−0.18	*0.451*	−0.58	*0.186*
Sensitive behavior	0.42	*0.557*	−0.24	*0.371*	0.21	*0.575*
Unresponsive behavior	0.17	*0.775*	−0.27	*0.231*	−0.71	*0.051*
Child exposure to violence	0.97	*0.427*	0.26	*0.560*	1.33	*0.062*
Alexithymia	0.11	*0.811*	−0.11	*0.555*	**0.73**	***0.042***
Maternal IPV-PTSD	0.30	*0.504*	0.21	*0.240*	**0.54**	***0.045***

*MDD, Major Depressive Disorder; IPV-PTSD, interpersonal violence related post-traumatic stress disorder; p, p-value*.

*p-values are in italic. Statistically significant results are in bold*.

*Continuous independent variables were standardized*.

*Model 1: unadjusted*.

*Model 2: adjusted for sex, age of the child, and maternal socio-economic status*.

*Model 3: model 2 further adjusted for maternal controlling behavior*.

*Model 4: model 3 further adjusted for child exposure to violence*.

*Model 5: model 4 further adjusted for maternal alexithymia*.

*Model 6: model 5 further adjusted for maternal IPV-PTSD*.

### Role of Maternal Caregiving Behaviors, Alexithymia, and Child Exposure to Family Violence and/or Treatment on the Association Between Maternal and Child Somatization and Psychopathology (Aim 3)

On further examination, we found that when the association was further adjusted for potential confounders such as maternal caregiving behaviors, alexithymia and child exposure to violence, the association between maternal somatization and number of MDD symptoms in children did not remain statistically significant. Additionally, our results showed an association between maternal somatization in phase 1 and maternal-reported child thought problems (e.g., obsessive thoughts, self-harm, hallucinations, sleep problems, strange behavior, nervous twitching) in Phase 2 even after adjusting the model for sociodemographic variables, maternal caregiving behaviors and child exposure to violence. However, the association did not remain statistically significant after further adjustment for maternal alexithymia. On the contrary, we found that maternal somatization in Phase 1 was associated with maternal-reported child somatization in Phase 2 after the fully adjusted models for potential confounders.

To understand better the relationship between maternal somatization with child somatization and thought problems, we performed additional analyses. Specifically, we tested whether maternal somatization was associated with an increase in maternal alexithymia, caregiving behaviors and child exposure to family violence, as well as the associations between maternal alexithymia, caregiving behaviors and child exposure to family violence with child somatization and thought problems. We found that maternal somatization was associated with a greater degree of maternal alexithymia in Phase 1 (β = 0.13, 95 CI [0.00–0.26], *p* = *0.045*. However, this association did not remain significant after adjustment for maternal age and SES (β = 0.13, 95 CI [−0.02 to 0.02], *p* = *0.062*). Similarly, maternal somatization was not statistically associated with maternal controlling behavior (β = 1.17, 95 CI [−0.08 to 2.43], and *p* = *0.066*), nor maternal unresponsive behavior (β = 0.45, 95 CI [−0.32 to 1.21], *p* = *0.245*), and child exposure to family violence (OR = 1.46, 95 CI [0.51–4.20], *p* = *0.478*). We did find, however, an association between maternal somatization and a decreased maternal sensitive behavior in Phase 1, after adjustment for maternal age and SES (β = −0.11, 95 CI [−0.21 to −0.02], *p* = *0.021*).

Moreover, maternal alexithymia in Phase 1 was associated with maternal-reported thought problems in children in Phase 2 (β = 0.11, 95 CI [0.03–0.18], *p* = *0.005*). We then evaluated the interaction between maternal somatization together with alexithymia on child somatization and thought problems. The two interactions did not reach statistical significance (β = 0.11, 95 CI [−0.56 to 0.79], *p* = *0.738* for child somatization; β = −0.19, 95 CI [−0.57 to 0.19], *p* = *0.318* for thought problems). We can therefore suppose that maternal alexithymia, caregiving behaviors and child exposure to violence do not play a role in the relationship between maternal and child somatization (assuming there is enough statistical power to detect such a difference). However, since alexithymia is apparently associated with both maternal somatization and thought problems in children, we hypothesize that alexithymia could be a confounder for the relationship between maternal somatization and maternal-reported thought problems in children.

## Discussion

**Figure 3:**
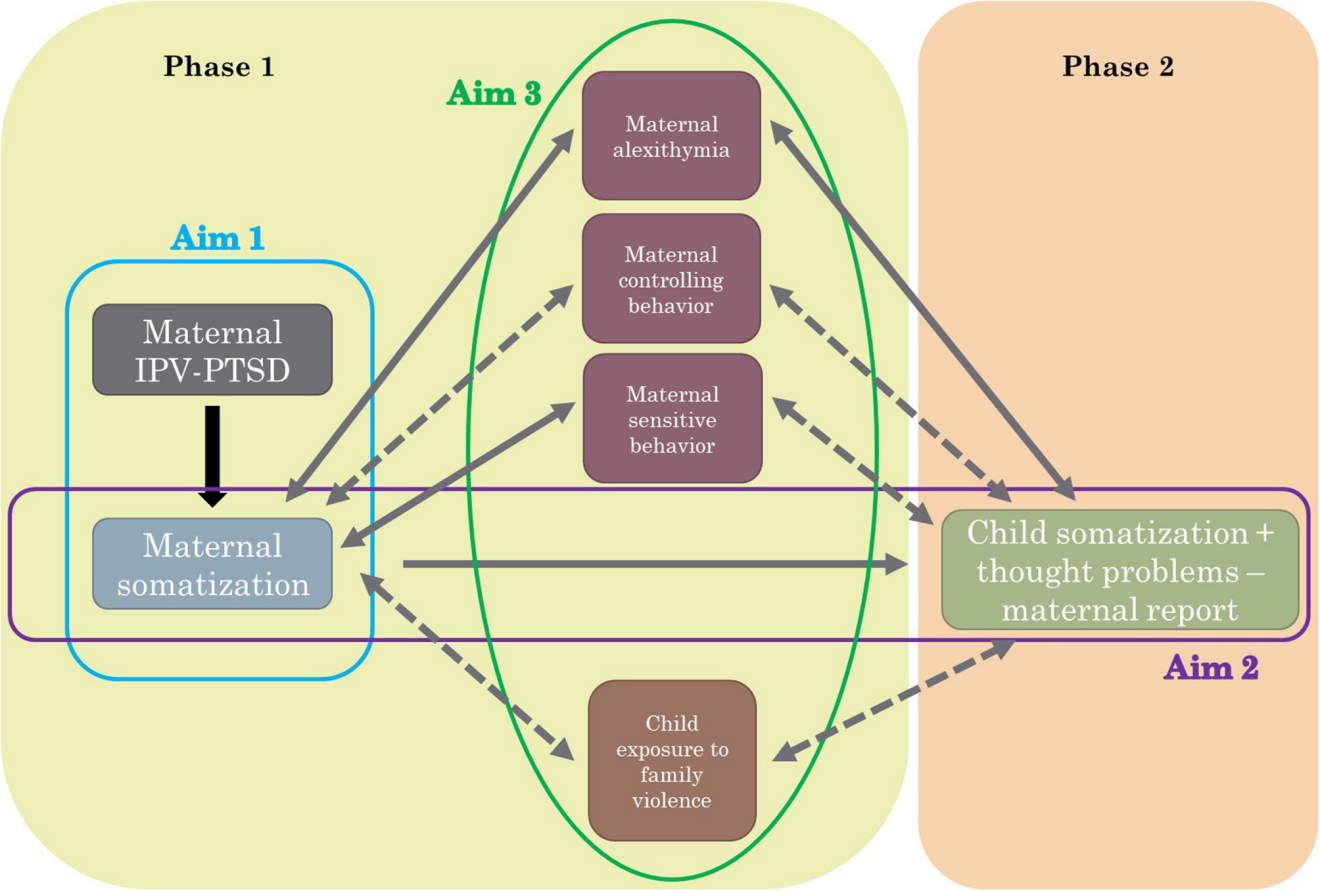
Summary of aims and main results. IPV-PTSD, interpersonal violence related PTSD. Aim 1: Association between maternal IPV-PTSD and maternal somatization in Phase 1. Aim 2: Association between maternal somatization in Phase 1 with child somatization in Phase 2. Aim 3: Evaluate the role of maternal alexithymia, controlling and sensitive behaviors and child exposure to family violence for the association between maternal and child somatization. Solid lines describe statistical significant associations, whereas dashed lines describe non-significant associations.

This study examined the intergenerational transmission of somatization while taking into account the history of exposure to familial interpersonal violence and related PTSD in a sample composed of mother-child dyads. We found an association between maternal somatization in Phase 1 with child somatization and thought problems by maternal report in Phase 2. Maternal alexithymia, caregiving behaviors, and child exposure to violence did not play a significant role in the association between maternal and child somatization at this study's level of analysis (see Figure 3 for a summary of the main results).

We have provided evidence for a significant association between maternal and child somatization severity by maternal report. Results suggest that this association may point to a form of intergenerational transmission of somatoform symptoms in the context of maternal post-traumatic stress related to interpersonal violence. We considered the possibility that maternal somatization might be a proxy for maternal PTSD and thus not be a distinct phenomenon in and of itself, such that it would be the PTSD carrying the effect. However, our analyses showed that the association between maternal and child somatization severity is, in fact, not accounted for by maternal PTSD. This is despite the fact that greater somatization was more common among mothers with PTSD than non-PTSD controls.

The literature supports that individuals with PTSD following experiences of childhood maltreatment and exposure to domestic violence, most often involving primary caregivers, display significantly more severe somatization than those without this comorbidity (68). However, additional measures related to IPV-PTSD (60), such as maternal alexithymia, the quality of caregiving behavior, and child exposure to violence (60), did not seem to significantly alter the association between maternal and child somatization in our study. One additional association of maternal somatization to child psychopathology was that of maternally reported child thought problems on the CBCL, such as morbid or aggressive ruminations, obsessions, and thoughts, including those contributing to nocturnal sleep disturbance. This subscale of thought problems was the only other category of child psychopathology besides child somatization that was associated with maternal somatization. No association was found between maternal somatization and child self-reported psychopathology.

While it was beyond the design of this study to determine the potential causality of the association between maternal and child somatization, the present study lacks genetic and other data that would make this possible. The literature, nevertheless, suggests that the association between maternal and child somatization and the caregiving environment as affected by early adversity within the context of attachment, may be linked (69). Maunder et al. (69) conducted a prospective, longitudinal study of 292 mother-infant dyads in two different lower adversity-risk control groups (ambulatory care patients both healthy and in remission from a chronic illness), and two different higher adversity risk groups (inpatient hospital health care providers and emergency room providers). That study found in the higher risk groups that mothers displayed less sensitive behavior with their infants at 6- and 18-months of age who displayed insecure attachment at 18-months of age. In turn, insecure attachment characterized by less sensitive maternal behavior was associated with significantly more somatization when the children were evaluated at age 5-years. A limitation of this latter study was the absence of measurement of maternal somatization, such as measured in the present study.

Nevertheless, we observed that only maternally reported psychopathology in children was associated with maternal somatization; whereas, child self-reported psychopathology was not associated with maternal somatization. Moreover, maternal-reported somatization in children was also not correlated with child self-reported psychopathology. Therefore, the association between maternal somatization and child somatization may well be the result of mothers' biased reporting. Mothers with a higher degree of somatization given their own somatic preoccupations, are more likely to focus selectively on, if not exaggerate, children's somatic complaints. The latter may thus represent over-reporting of child symptoms by mothers who themselves experience greater unmentalized psychological distress, as one study suggested (70). “Mentalization,” operationalized as “reflective functioning” as a quantitative research measure, in the parent is an important attachment-related correlate, if not predictor, of maternal sensitive caregiving (61). Mentalization refers to the ability of the individual to infer and represent mental states of the self and other and to link those mental states to actions (71). The literature makes the link between childhood maltreatment by an attachment figure and decreased capacity for mentalization, leading to an over-emphasis on somatic expressions of distress, thus rendering intergenerational transmission both of maltreatment, emotion dysregulation, and somatization more likely in this context of reduced parental mentalization (41, 61). Indeed, one study demonstrated that mothers who report chronic pain, are more likely to be focused on their own and others' somatic discomfort, rather than their own or others' mental states, and in so doing be more likely to confer risk for somatoform disorders to their children (72). Additionally, mothers who experience and express more negative affect and cognitions have been found to report higher levels of child somatic symptoms (73). Indeed, individuals with complex PTSD such as the violence-exposed mothers with PTSD in the present study, have a tendency to express more negative affect and cognitions (74). Whether or not what we suggest is likely a maternal selective attention, if not exaggeration of child somatic complaints, has an impact on adolescent or adult development of psychopathology in these children, is a question that remains beyond the scope of the present study.

That being said, an alternative hypothesis is that this finding could also represent a tendency toward underreporting of psychopathological symptoms among children who actually do experience them, as was found in one study of adolescents who suffered from a chronic medical illness, who underreported their somatic symptoms (75). However, this is only a speculation. To understand which of these latter two possibilities (i.e., over-reporting by mothers, or under-reporting by the children) is more likely, future research within a similar population might best use a child self-reported measure specifically of somatization.

In any case, the pertinent literature suggests that somatization among some individuals represents either a deficit or possibly a psychological defense that impedes the mental representation of psychological distress and psychic conflict, both affectively and/or cognitively (76, 77). It is important to note, for clinicians treating children, that parents who report higher levels of somatization such as the mothers we have described in this paper, may well have difficulties in this area of psychological mindedness when caring for young children. And these difficulties may well affect their child's development in more subtle ways, in terms of skills of self-regulation, social cognition, and in particular, mentalization (78), as well as abstract thinking as puberty approaches (79). One prospective, longitudinal study has indeed shown that family conflict and difficulty in expressing emotions during early childhood and school age predict greater somatization during adolescence (80, 81). Although a previous study found that maternal withdrawal and emotional unavailability was the most salient predictor of child somatization (82), we did not find this in our study. The inconsistent result could be due again to the fact that child somatization was reported by the mother, and therefore this could be biased. However, we also found an association between maternal alexithymia and child somatization, which partially goes in the same direction as the previous studies mentioned.

An additional link between early exposure specifically and interpersonal violence exposure (i.e., physical and sexual abuse and domestic violence) may be salient in light of effects on cognitive-affective development across school age; namely, the association with thought problems in children. This latter association between maternal somatization and child thought problems may be considered in light of both the failure to extinguish traumatic memory traces following victimization during sensitive periods for the development of security of bodily integrity, and the need to dissociate or split off the strong negative emotions associated with betrayal by one's caregiver (83, 84). Moreover, families in which maltreatment and violence occur tend to have lower levels of education, which is an association supported by the present study in terms of difference in socio-economic status of which the number of years of maternal education plays an important role (29, 30, 48). Additionally, we noted in a controlled study that mothers with histories of early adversity who develop PTSD manifest a significantly lower capacity for reflective functioning that is associated with an altered pattern of neural activity in response to video excerpts showing a high-stress relational interaction (separation) vs. a low-stress interaction (free-play) (61, 85).

The finding of an association of maternal somatization with maternal-reported child thought problems including obsessive thoughts, ruminations, sleep disturbances, odd thoughts, and sensations on a spectrum toward frank psychotic symptoms, was of clinical interest in that it may be that children of somatizing mothers have more difficulty processing their day-to-day experiences with a primary attachment figure who is able to help the child think about and regulate their emotions with respect to those experiences. Indeed, after controlling for maternal alexithymia, this association of maternal somatization and child thought problems was no longer significant. The presence of maternal alexithymia together with IPV-PTSD, we had found, was associated with less sensitive and more controlling/frightening, unresponsive/frightened and otherwise atypical caregiving behavior (60). Therefore, the notion that longitudinally, at school age, this combination also poses a risk factor, at least for maternal report of child thought problems on the CBCL, merits further study. Indeed, the association of maternal somatization to alexithymia did not remain significant after adjusting for maternal SES. This points also to a link between the capacity to identify emotions in self and other and perhaps the reliance on somatization as a non-psychological means of expressing psychological distress, precisely to fewer years of maternal education. This, however, remains an association that may well be bidirectional, and likely more complex than it initially appears (86). Thus, it can be difficult to tease apart how less education may encourage psychological dysfunction, and how psychological dysfunction running in families may lead to less motivation and/or greater difficulty in pursuing higher education (87). However, as already exposed above, child thought problems were reported by the mothers. Therefore, we could also make the assumption that maternal report is biased.

### Limitations

This study has a number of important limitations, foremost of which is the absence of a clinical interview of the children that captures somatization and that would provide greater information as to the specific nature of child somatization. While parental report of child somatization tends to be fairly consistent with child report (88), total reliance on maternal report for this area of psychopathology that is crucial to this paper is a limitation. Moreover, other forms of child psychopathology were measured *via* a semi-structured interview administered only to the children and not with parent collateral report. As such, we report only the measure of child-reported symptoms. Diagnoses were based on clinician judgment with this in mind, rather than *via* the completed interview. Maternal trauma history was retrospectively self-reported, which can lead to a margin of error and distortion (89). However, converging findings with maternal psychopathology suggest that this is a relatively minor limitation. Additionally, given the complexity of estimating the statistical power of moderated multiple regressions (90), it cannot be excluded that, despite our power analysis, interaction effects between maternal somatization and alexithymia on some of our findings may elude a sample of the present studies' size. Future studies should test the interactions in larger samples.

### Clinical Implications

The identification of a potential intergenerational pattern of somatization in the context of maternal early adversity that is characteristic of maternal functioning on a stable basis beginning during her child's earliest and most formative development suggests the importance of early infant-parent intervention. Such intervention may increase mothers' ability to represent their own and their child's psychological distress, and possible psychic conflicts, for example of approach/avoidance to abusive or otherwise violent caregivers, in mental states that they can describe in language. Interventions that support and model parental reflective functioning (91) may provide a foundation for more adaptive psychological functioning across generations. Additional forms of psychotherapy that encourage mind-body connection, such as arts therapies, mindfulness-based therapies may provide additional tools that address the mind-body split that seems to underlie somatization and somatoform disorders.

## Conclusion

Our results are in line with the hypothesis of the intergenerational transmission of somatization in the context of interpersonal violence and related maternal PTSD during formative early development. We interpret the higher rate and severity of somatization among the post-traumatically stressed mothers as increasing the risk of maternal bias toward identifying somatic rather than psychological distress in their children, as they do in themselves. This focus, together with findings reported in other papers by the authors, affect the caregiving environment during sensitive periods of social and emotional development, particularly that of mentalization. This finding thus suggests the importance of early infant-parent intervention. Future studies should use a child self-reported measure of somatization, in order to understand whether mothers with PTSD tend to over-report, or their children tend to under-report child somatization.

## Data Availability Statement

The raw data supporting the conclusions of this article will be made available by the authors, without undue reservation.

## Ethics Statement

The studies involving human participants were reviewed and approved by Institutional Ethics' Committee of the Geneva University Hospitals. Written informed consent to participate in this study was provided by the participants' legal guardian/next of kin.

## Author Contributions

JG undertook the main statistical analysis, contributed to the interpretation of the data, and drafted the manuscript. DM contributed to the interpretation of the data and critically revised the drafts for important intellectual content. SR, SJ, FT, and KP critically revised the drafts for important intellectual content. DS designed the study, supervised data collection, data management and the statistical analysis, contributed to the interpretation of the data, and critically revised the drafts. All authors contributed to the article and approved the submitted version.

## Funding

This study was funded by a Swiss National Science Foundation NCCR-SYNAPSY grant (n° 51AU40_125759).

## Conflict of Interest

The authors declare that the research was conducted in the absence of any commercial or financial relationships that could be construed as a potential conflict of interest.

## Publisher's Note

All claims expressed in this article are solely those of the authors and do not necessarily represent those of their affiliated organizations, or those of the publisher, the editors and the reviewers. Any product that may be evaluated in this article, or claim that may be made by its manufacturer, is not guaranteed or endorsed by the publisher.
